# Supplementary material for: COZOID: contact zone identifier for visual analysis of protein-protein interactions
Source: BMC Bioinformatics. 2018 Apr 6;19:125. doi: 10.1186/s12859-018-2113-6 (PMC5889581; doi:10.1186/s12859-018-2113-6)
Supplement: Supplementary file 2 — Software build. Executable binary file of the software tool. (ZIP 118,784 kb) [file 12859_2018_2113_MOESM2_ESM.zip › cozoid/java/docs/beansbinding-1.2.1-doc.zip!/serialized-form.html]

Serialized Form (Beans Binding)


---


|  |  |  |
| --- | --- | --- |
| **Overview** \| Package \| Class \| Use \| **Tree** \| **Deprecated** \| **Index** \| **Help** | |  |
| PREV   NEXT | **FRAMES**    **NO FRAMES**     **All Classes** |  |


---


# Serialized Form


---

**Package** **org.jdesktop.beansbinding**

**Class org.jdesktop.beansbinding.PropertyResolutionException extends java.lang.RuntimeException implements Serializable**

**Class org.jdesktop.beansbinding.PropertyStateEvent extends java.util.EventObject implements Serializable**

**Serialized Fields**

### sourceObject

```
java.lang.Object sourceObject
```

---

### valueChanged

```
boolean valueChanged
```

---

### oldValue

```
java.lang.Object oldValue
```

---

### newValue

```
java.lang.Object newValue
```

---

### writeableChanged

```
boolean writeableChanged
```

---

### isWriteable

```
boolean isWriteable
```

---

**Package** **org.jdesktop.el**

**Class org.jdesktop.el.ELContextEvent extends java.util.EventObject implements Serializable**

**Class org.jdesktop.el.ELException extends java.lang.RuntimeException implements Serializable**

**Class org.jdesktop.el.Expression extends java.lang.Object implements Serializable**

**Class org.jdesktop.el.MethodExpression extends Expression implements Serializable**

**Class org.jdesktop.el.MethodNotFoundException extends ELException implements Serializable**

**Class org.jdesktop.el.PropertyNotFoundException extends ELException implements Serializable**

**Class org.jdesktop.el.PropertyNotWritableException extends ELException implements Serializable**

**Class org.jdesktop.el.ValueExpression extends Expression implements Serializable**

**Serialized Fields**

### source

```
java.lang.Object source
```

---

**Package** **org.jdesktop.el.impl**

**Class org.jdesktop.el.impl.MethodExpressionImpl extends MethodExpression implements Serializable**

**Serialization Methods**

### readExternal

```
public void readExternal(java.io.ObjectInput in)
                  throws java.io.IOException,
                         java.lang.ClassNotFoundException
```

:   **Throws:**: `java.io.IOException`: `java.lang.ClassNotFoundException`

---


### writeExternal

```
public void writeExternal(java.io.ObjectOutput out)
                   throws java.io.IOException
```

:   **Throws:**: `java.io.IOException`

**Class org.jdesktop.el.impl.MethodExpressionLiteral extends MethodExpression implements Serializable**

**Serialization Methods**

### readExternal

```
public void readExternal(java.io.ObjectInput in)
                  throws java.io.IOException,
                         java.lang.ClassNotFoundException
```

:   **Throws:**: `java.io.IOException`: `java.lang.ClassNotFoundException`

---


### writeExternal

```
public void writeExternal(java.io.ObjectOutput out)
                   throws java.io.IOException
```

:   **Throws:**: `java.io.IOException`

**Class org.jdesktop.el.impl.ValueExpressionImpl extends ValueExpression implements Serializable**

**Serialization Methods**

### readExternal

```
public void readExternal(java.io.ObjectInput in)
                  throws java.io.IOException,
                         java.lang.ClassNotFoundException
```

:   **Throws:**: `java.io.IOException`: `java.lang.ClassNotFoundException`

---


### writeExternal

```
public void writeExternal(java.io.ObjectOutput out)
                   throws java.io.IOException
```

:   **Throws:**: `java.io.IOException`

**Class org.jdesktop.el.impl.ValueExpressionLiteral extends ValueExpression implements Serializable**

**serialVersionUID:** 1L

**Serialization Methods**

### readExternal

```
public void readExternal(java.io.ObjectInput in)
                  throws java.io.IOException,
                         java.lang.ClassNotFoundException
```

:   **Throws:**: `java.io.IOException`: `java.lang.ClassNotFoundException`

---


### writeExternal

```
public void writeExternal(java.io.ObjectOutput out)
                   throws java.io.IOException
```

:   **Throws:**: `java.io.IOException`

---

**Package** **org.jdesktop.el.impl.lang**

**Class org.jdesktop.el.impl.lang.FunctionMapperImpl extends FunctionMapper implements Serializable**

**serialVersionUID:** 1L

**Serialization Methods**

### readExternal

```
public void readExternal(java.io.ObjectInput in)
                  throws java.io.IOException,
                         java.lang.ClassNotFoundException
```

:   **Throws:**: `java.io.IOException`: `java.lang.ClassNotFoundException`

---


### writeExternal

```
public void writeExternal(java.io.ObjectOutput out)
                   throws java.io.IOException
```

:   **Throws:**: `java.io.IOException`

**Class org.jdesktop.el.impl.lang.FunctionMapperImpl.Function extends java.lang.Object implements Serializable**

**Serialization Methods**

### readExternal

```
public void readExternal(java.io.ObjectInput in)
                  throws java.io.IOException,
                         java.lang.ClassNotFoundException
```

:   **Throws:**: `java.io.IOException`: `java.lang.ClassNotFoundException`

---


### writeExternal

```
public void writeExternal(java.io.ObjectOutput out)
                   throws java.io.IOException
```

:   **Throws:**: `java.io.IOException`

**Class org.jdesktop.el.impl.lang.VariableMapperImpl extends VariableMapper implements Serializable**

**serialVersionUID:** 1L

**Serialization Methods**

### readExternal

```
public void readExternal(java.io.ObjectInput in)
                  throws java.io.IOException,
                         java.lang.ClassNotFoundException
```

:   **Throws:**: `java.io.IOException`: `java.lang.ClassNotFoundException`

---


### writeExternal

```
public void writeExternal(java.io.ObjectOutput out)
                   throws java.io.IOException
```

:   **Throws:**: `java.io.IOException`

---

**Package** **org.jdesktop.el.impl.parser**

**Class org.jdesktop.el.impl.parser.ParseException extends java.lang.Exception implements Serializable**

**Serialized Fields**

### specialConstructor

```
boolean specialConstructor
```

:   This variable determines which constructor was used to create
    this object and thereby affects the semantics of the
    "getMessage" method (see below).

---

### currentToken

```
Token currentToken
```

:   This is the last token that has been consumed successfully. If
    this object has been created due to a parse error, the token
    followng this token will (therefore) be the first error token.

---

### expectedTokenSequences

```
int[][] expectedTokenSequences
```

:   Each entry in this array is an array of integers. Each array
    of integers represents a sequence of tokens (by their ordinal
    values) that is expected at this point of the parse.

---

### tokenImage

```
java.lang.String[] tokenImage
```

:   This is a reference to the "tokenImage" array of the generated
    parser within which the parse error occurred. This array is
    defined in the generated ...Constants interface.

---

### eol

```
java.lang.String eol
```

:   The end of line string for this machine.

**Class org.jdesktop.el.impl.parser.TokenMgrError extends java.lang.Error implements Serializable**

**Serialized Fields**

### errorCode

```
int errorCode
```

:   Indicates the reason why the exception is thrown. It will have
    one of the above 4 values.

---


|  |  |  |
| --- | --- | --- |
| **Overview** \| Package \| Class \| Use \| **Tree** \| **Deprecated** \| **Index** \| **Help** | |  |
| PREV   NEXT | **FRAMES**    **NO FRAMES**     **All Classes** |  |


---
